# Better Survival with Three-Dimensional Conformal Radiotherapy Than with Conventional Radiotherapy for Cervical Cancer: A Population-Based Study

**DOI:** 10.1155/2013/729819

**Published:** 2013-10-02

**Authors:** Chen-Hsi Hsieh, Shiang-Jiun Tsai, Wen-Yen Chiou, Moon-Sing Lee, Hon-Yi Lin, Shih-Kai Hung

**Affiliations:** ^1^Department of Radiation Oncology, Far Eastern Memorial Hospital, Taipei 220, Taiwan; ^2^Department of Medicine, School of Medicine, National Yang-Ming University, Taipei 112, Taiwan; ^3^Institute of Traditional Medicine, School of Medicine, National Yang-Ming University, Taipei 112, Taiwan; ^4^Department of Radiation Oncology, Buddhist Dalin Tzu Chi General Hospital, Chiayi 622, Taiwan; ^5^School of Medicine, Tzu Chi University, Hualien 970, Taiwan

## Abstract

Three-dimensional conformal radiation therapy (3DCRT) has emerged as a preferred treatment for gynecologic malignancies. Yet its superiority to conventional radiotherapy (2-dimensional radiotherapy (2DRT)) for gynecologic malignancies has not been well established. Data from the 2005 to 2010 National Health Insurance Research Database (NHIRD) provided by the National Research Institutes in Taiwan were analyzed to address this issue. Patients were initially diagnosed as having cervical cancer according to the International Classification of Disease, Ninth Revision, Clinical Modification (ICD-9-CM) code 180, and this clinical diagnosis was confirmed histopathologically or cytologically. Kaplan-Meier method and Cox proportional hazards regression were used to analyze the reported data. Between January 2005 and December 2010, there were 776 patients with newly diagnosed cervical cancer without metastasis, local recurrence, or surgical treatment before RT and 132 and 644 patients, respectively, who received 2DRT and 3DCRT. After adjustment for age, diabetes mellitus, hypertension, coronary heart disease, hyperlipidemia, side effects, urbanization level, geographic region, and enrollee category in the 5-year follow-up period, the HR was 1.82 (95% CI, 1.16–2.85, *P* = 0.009). The 5-year survival rate in the 2DRT and 3DCRT groups was 73.0% and 82.3%, *P* = 0.007, respectively. Cervical cancer patients treated with 3DCRT had better overall survival.

## 1. Introduction

Cervical cancer is the second most frequent cancer among women worldwide and the most frequent cancer among women in Africa, Asia, and South America [1]. Concurrent chemotherapy with external beam radiotherapy (EBRT) shows benefit for patients with bulky and locally advanced cervical cancer [2–5]. Though dose is related to local control and overall survival, the risk of tissue toxicity (acute or late) currently limits the total radiation dose that can safely be delivered [6, 7]. Risk factors for morbidity include the volume of irradiated normal tissue, total tumor dose, EBRT dose, fraction size, and age [8–10]. These factors can lead to unplanned treatment breaks and long overall treatment times that may negatively influence the outcome. Therefore, dose escalation, decreasing toxicity to normal tissues, and the use of novel systemic agents have tremendous potentials to improve the outcome.

Conventional radiotherapy (2-dimensional radiotherapy (2DRT)) uses bony landmarks to define the target volume for pelvic radiotherapy. Treatment is delivered either with anterior and posterior opposed fields or with a four-field box technique, which reduces the volume of small bowel in the treated volume [11, 12]. However, studies assessing the adequacy of the standard fields for target volume coverage have reported an underdosing of the designated lymph node regions in 30–40% of patients [13–15]. In the late 1990s, the technique of three-dimensional conformal radiation therapy (3DCRT) emerged as a preferred treatment for gynecologic malignancies, since it gave better and more precise target coverage (20% reduction in the risk of a geographical miss) and significantly reduced the volume of radiation-exposed bladder and bowel [16, 17]. These results are consistent with the findings of reduced dose to normal structures such as the gastrointestinal tract (32% and 19% grade 2 toxicity of 3DCRT and 2DRT, resp., for prostate cancer; *P* = 0.02) [18].

The proven benefits of 3DCRT over the 2DRT technique have led to studies to determine whether 3DCRT is superior to 2DRT for the clinical treatment of gynecologic malignancies. To prove the superiority of 3DCRT, a large-scale, nationwide, controlled cohort study was conducted in Taiwan to investigate the relative benefits of 3DCRT and 2DRT in patients with gynecologic malignancies. 

## 2. Materials and Methods

### 2.1. Ethics Statement

The study protocol was approved by the Buddhist Dalin Tzu Chi General Hospital Institutional Review Boards. Informed consent was not needed and waived because only deidentified retrospective data released to the public for research was collected and analyzed.

### 2.2. Database

The study analyzed data from the 2005 to 2010 National Health Insurance Research Database (NHIRD) provided by the National Research Institutes in Taiwan. The National Health Insurance program was implemented in Taiwan in 1995. The database contains comprehensive information on insured subjects, including dates of clinical visits, diagnostic codes, details of prescriptions, and expenditure amounts. The NHIRD contains the medical benefit claims for 97% of the population and a registry of board-certified physicians and contracted medical facilities.

### 2.3. Study Population

**Figure 1:**
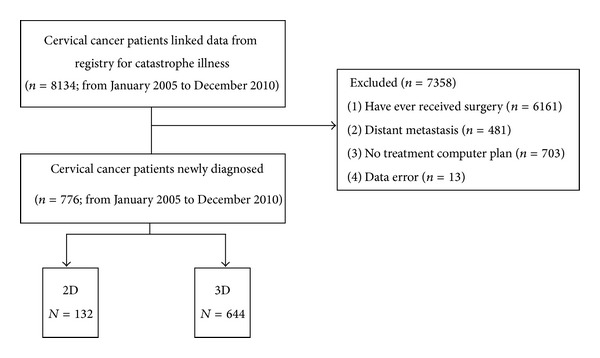
A flowchart of this population-based study showing the selection and group allocation of the cohort used for analysis.

Patients were initially identified as having cervical cancer according to the International Classification of Disease, Ninth Revision, Clinical Modification (ICD-9-CM) code 180, and then, the clinical diagnosis was validated using the cross-linked data from the registry for catastrophic illness (*n* = 33, 205). Next, we identified 8134 patients with cervical cancer newly diagnosed between January 2005 to December 2010. From this patient cohort, we excluded patients with distant metastases at the time of diagnosis (*n* = 481), prior surgery (*n* = 6161), no treatment plan (*n* = 703), and errors in coded data (*n* = 13). Finally, we identified and divided 776 cervical cancer patients receiving radiotherapy into two study groups: one receiving 2DRT (*n* = 132) and the other 3DCRT (*n* = 644). The flow diagram in Figure 1 shows the allocation of patients to the two study groups.

### 2.4. Measurements

The key dependent variable of interest was the 5-year survival rate. Survival was measured from the time of cervical cancer diagnosis to the time of death. The independent variables were age, comorbidities, side effects, geographic region, urbanization level, and socioeconomic status. Comorbidities included hypertension (ICD-9-CM codes 401–405), diabetes (ICD-9-CM code 250), coronary heart disease (ICD-9-CM codes 410–414), and hyperlipidemia (ICD-9-CM codes 2720–2724). Diarrhea or radiation proctitis, gastroenteritis, and colitis due to radiation (ICD-9-CM code 5581), radiation cystitis, or overactive bladder (ICD-9-CM codes 59582, 59589, and 59651), dermatitis due to other radiation (ICD-9-CM code 69282), other myelopathy (ICD-9-CM code 3368), late effect of radiation (ICD-9-CM code 9092), and other unspecific radiation side effects (ICD-9-CM code 990) were defined as side effects. There were five geographic regions (northern, central, southern, eastern, and other) and four urbanization levels (urban, suburban, rural, and other). This study also used enrollee category (EC) in the NHIRD as a proxy measure of socioeconomic status. All patients were divided into 4 subgroups: EC 1 (civil servants, full-time or regular paid personnel with a government affiliation), EC 2 (employees of privately owned institutions), EC 3 (self-employed individuals, other employees, and members of farmers' or fishermens' associations), and EC 4 (veterans, low-income families, and substitute service draftees).

### 2.5. Statistical Analysis

The statistical software packages SAS (version 9.2; SAS Institute, Inc., Cary, NC, USA) and SPSS (version 17; SPSS Inc., Chicago, IL, USA) were used for data analysis. Pearson's chi-square tests were used to explore the differences between categorical variables in the different plan groups. The 5-year survival rate was estimated using the Kaplan-Meier method and compared by the logrank test. A Cox proportional hazard regression model was used to calculate the relative risk of cervical cancer patients between different plan modalities after adjusting for age, diabetes mellitus, hypertension, coronary heart disease, hyperlipidemia, side effects, residence urbanization level, and socioeconomic status. A *P* < 0.05 was defined as statistically significant. 

## 3. Results

**Figure 2:**
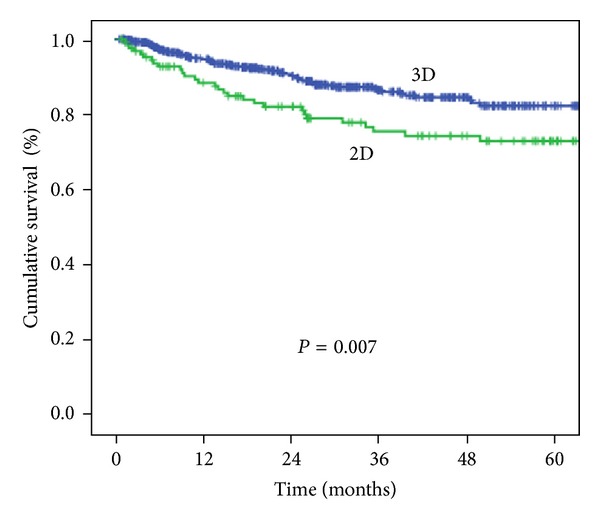
Cumulative survival of the 2DRT and 3DCRT groups from 2005 to 2010.

**Figure 3:**
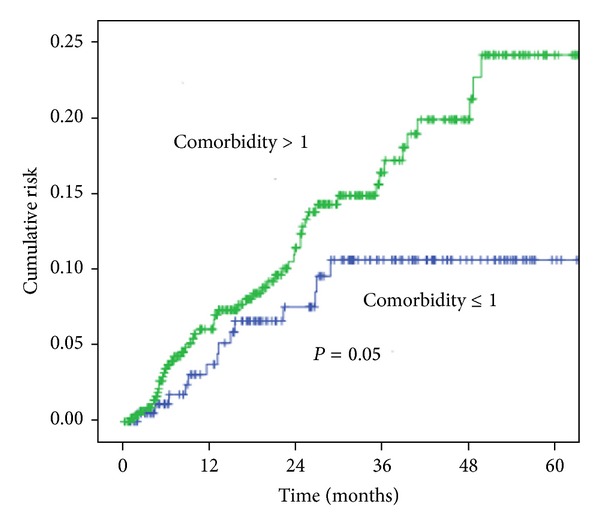
The survival of cervical cancer patients with >1 risk factor compared with that in patients ≤1 risk factor.

**Figure 4:**
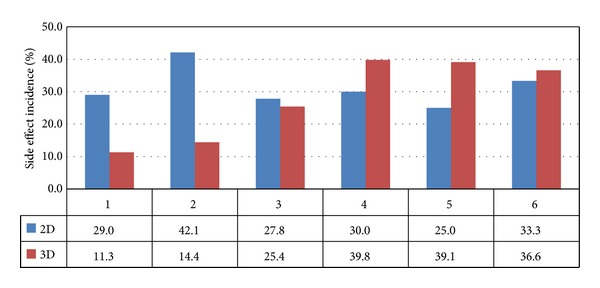
Annual incidence trend of side effect postradiotherapy. *X axis means interval year of postradiotherapy; *Y* axis means side effect incidence (%).

The characteristics and comorbidities of the patients included in the study are shown in Table 1. After adjustment for age, diabetes mellitus, hypertension, coronary heart disease, hyperlipidemia, side effects, urbanization level, geographic region, and EC in the 5-year follow-up period, HR was 1.82 (95% CI, 1.16–2.85, *P* = 0.009) (Table 2). Five-year survival rate for 2DRT and 3DCRT was significantly different (73.0% and 82.3%, resp., *P* = 0.007; Figure 2). Additionally, age older than 55 years, hypertension, diabetes, coronary heart disease, and hyperlipidemia were used to stratify the cervical cancer patients into a low-risk group (*n* = 221) and high-risk group (*n* = 555). The 5-year cumulative survival rate for the two-risk groups was 86.5% (3DCRT) and 78.0% (2DRT; *P* = 0.05; Figure 3). The incidence trend of side effect after 2DRT and 3DCRT was showed in Figure 4.

## 4. Discussion

Optimal nodal coverage is critical in the treatment of cervical cancer, and RT has been shown to sterilize nodes. Moreover, pelvic failure is associated with decreased survival [19, 20]. 2DRT consists of a single beam from one to four directions. Beam setups are usually quite simple; plans frequently consist of opposed lateral fields or four-field “boxes” and use bony landmarks to confirm. However, use of bony landmarks is associated with a degree of uncertainty. The inadequacy of the standard fields for target volume coverage and underdosing in lymph node regions in around 30–40% of patients has been reported [13–15]. Kim [21] noted that margins are inadequate in 39–50% of cases. Russell et al. [22] also noted that the rate of missed therapeutic margins and the rate of incomplete coverage of the uterine fundus are as high as 24% and 62.5%, respectively. Even the use of 4-field radiation to the pelvis was potentially dangerous without a CT scan to confirm. The incidence of inadequate margin ranged from 39% to 50% and was independent of the stage of the disease, and the most common site of inadequate margin was the rectum [21]. Therefore, defining the lymph node structures to be included in the treatment plan and radiotherapy target volume is an important issue for cervical cancer patients. 

To confirm the area of lymph node targeting in 2DRT, direct visualization with lymphangiography (LAG) has been suggested [13]. Pendlebury et al. [23] using lymphangiography reported that a margin of 2.5 cm lateral to the pelvic sidewall would be required to cover the pelvic lymph nodes in 90% of patients. In their analysis, the superior border of the anterior-posterior (AP)/PA portals in 14% of patients has to be altered from the L5/S1 junction to L4/L5 to cover the common iliac nodes. Since computed tomography (CT) scans have become available in many radiotherapy departments, several attempts to improve treatment planning have been made by taking into account the anatomy of individual patients. Because sectional CT enables the visualization and delineation of the cervix, uterus, vagina, iliac vessels, and organs at risk such as bladder, rectum, and intestine, 3DCRT has become a preferred treatment for gynecologic malignancies. It gives better, more precise target coverage while reducing the risk of a geographical miss by 20% [16]. Although 4-field radiation technique spares the small bowel anteriorly and a portion of the rectum posteriorly, it is potentially dangerous to use the 4-field pelvic technique without knowledge of the precise tumor volume. Therefore, Kim [21] strongly recommended CT treatment planning. With more targeted treatment, better results can be predicted. These characteristics resulted in improved overall survival in our study. The survival rate was better in our 3DCRT group than in our 2DRT group (82.3% and 73.0%, *P* = 0.05) (Figure 1).

In the study by Souhami et al. [24] using conventional concurrent chemoradiation therapy to treat cervical cancer followed by high dose brachytherapy, the rate of response was high but severe late gastrointestinal complications developed in 28% of 50 patients. In the phase 3 study by Rose et al. [2], 38% of patients treated with 2DRT concurrent with cisplatin experienced grade 3 and 4 toxicities and 35% of patients treated with 2DRT + cisplatin for bulky stage IB cervical cancers experienced grade 3 or grade 4 adverse effects [25]. In the recent update of the Radiation Therapy Oncology Group (RTOG) 90-01 trial, 12% of patients treated with extended-field radiotherapy for common iliac or para-aortic lymph node involvement had late grade 3 and 4 toxicities [26]. In the RTOG 79-02 report, the risk of grade 4 and 5 toxicities doubled with extended-field treatment compared with involved-field treatment [27]. The addition of concurrent chemotherapy to extended-field radiotherapy magnifies the acute toxicity. The grade 3 and 4 acute bowel toxicity was 49% with conventional delivery of extended-field radiotherapy via opposed AP/PA fields to the para-aortic lymph nodes [28]. Furthermore, as the focus of extended treatment can encompass a large volume of bone marrow [29], potential hematologic depression could lead to untoward treatment interruptions, reducing the number and intensity of chemotherapy cycles [30]. 

Studies have shown that 3DCRT improves patient tolerance to curative treatment and allows for dose escalation [31]. Gerstner and colleagues reported that 3DCRT (compared with 2DRT) significantly reduces the volume of radiation exposure in the bladder (up to 34%) and bowel (up to 254 cm^3^) of cervical cancer patients [16]. Additionally, 3DCRT (compared with 2DRT) also decreases the dose to the small bowel up to 33% in postoperative node-positive cervical cancer patients [17]. Similarly, Hanks et al. [32] showed that conformal RT (compared with standard techniques of external beam therapy) decreased RTOG-EORTC grade 2 acute morbidity in prostate cancer patients. In other studies, the use of 3D planning for the entire course of treatment, rather than just the last part of the treatment, reduced the incidence of gastrointestinal complications [33, 34]. These results were consistent with dose reduction to normal structures in prostate cancer patients treated by 3DCRT (19% and 32% grade 2 gastrointestinal toxicity for 3DCRT and 2DRT, resp., *P* = 0.02) [18]. In the current study, side effects of 3DCRT were significantly lower than those of 2DRT (31% versus 23%, *P* = 0.04). This could be one reason why survival rate was better in the 3DCRT treatment group.

This analysis has several limitations. First, the diagnosis of cervical cancer, and any other comorbid conditions, was completely dependent on ICD codes. Nonetheless, the Bureau of National Health Insurance in Taiwan randomly reviews records and interviews patients to verify the accuracy of the diagnosis [35]. Second, cancer stages were not considered because this information was not available from the database. Instead of cancer-specific survival rate, overall survival rate was used, because the former could not be determined from this registry data. Third, several studies have reported that diabetes mellitus could be a factor influencing survival [36, 37], and the rate of diabetes mellitus in the current study was 31% in the 2DRT group and 21% in the 3DRCT group (*P* = 0.01). However, after adjustment for diabetes, the hazard ratio for the two groups was still significantly different (HR = 1.82, *P* = 0.009), suggesting that the influence of diabetes on overall survival could be less important.

In summary, 3DCRT (compared with 2DRT) leads to a better overall survival rate in cervical cancer patients. Cervical cancer patients with more comorbidities have poorer survival rate. Strategies to reduce the risk of comorbidity should be assessed. 

## Figures and Tables

**Table 1:** Demographic characteristics and comorbidities of cervical cancer patients in the 2D and 3D groups.

	2D		3D		*P *value
	(*n* = 132)		(*n* = 644)		
	*n*	%	*n*	%	
Age (in years)					0.67
0–44	16	12.1	83	12.9	
45–54	32	24.2	183	28.4	
55–64	36	27.3	152	23.6	
65–74	33	25.0	139	21.6	
75+	15	11.4	87	13.5	
Diabetes mellitus					0.01
Yes	41	31.1	138	21.4	
No	91	68.9	506	78.6	
Hypertension					0.75
Yes	54	40.9	273	42.4	
No	78	59.1	371	57.6	
Coronary heart disease					0.13
Yes	35	26.5	133	20.7	
No	97	73.5	511	79.3	
Hyperlipidemia					0.13
Yes	41	31.1	160	24.8	
No	91	68.9	484	75.2	
Side effects					0.04
Yes	41	31.1	148	23.0	
No	91	68.9	496	77.0	
Urbanization level					0.18
Urban	27	20.5	189	29.3	
Suburban	65	49.2	270	41.9	
Rural	39	29.5	177	27.5	
Other	1	0.8	8	1.2	
Geographic region					0.60
Northern	50	37.9	289	44.9	
Central	49	37.1	197	30.6	
Southern	3	2.3	14	2.2	
Eastern	29	22.0	139	21.6	
Other	1	0.8	5	0.8	
EC					0.06
EC 1, 2	32	24.2	111	17.2	
EC 3	50	37.9	221	34.3	
EC 4	20	15.2	96	14.9	
Other	30	22.7	216	33.5	

EC indicates enrollee category.

**Table 2:** Crude and adjusted hazard ratio for the two groups in the 5-year follow-up period.

	Event %	Unadjusted HR	*P *value	Adjusted HR	*P *value
		(95% CI)		(95% CI)	
Cervical cancer patients with 3D treatment (*n* = 644)	67 (10.4)	1	0.008	1	0.009
Cervical cancer patients with 2D treatment (*n* = 132)	29 (22.0)	1.80 (1.16–2.79)		1.82 (1.16–2.85)	

Adjusted for age, diabetes mellitus, hypertension, coronary heart disease, hyperlipidemia, side effects, urbanization level, geographic region, and enrollee category.
